# Mediating Effect of Psychological Distress and Sexual Satisfaction in Puerto Ricans with Prostate Cancer

**DOI:** 10.37226/rcp.v4i3.4953

**Published:** 2020

**Authors:** Carmen T. Otero-Cordero, Coralee Pérez-Pedrogo, Adam Rosario-Rodríguez

**Affiliations:** 1 Carlos Albizu University, San Juan, Puerto Rico; 2 Carlos Albizu University, San Juan, Puerto Rico; 3 Carlos Albizu University, San Juan, Puerto Rico

**Keywords:** emotional distress, erectile dysfunction, quality life, sexual satisfaction, angustia psicológica, disfunción eréctil, calidad de vida, satisfacción sexual

## Abstract

Prostate cancer is the most diagnosed in the male population in Puerto Rico. However, it is little studied in the field of health, specifically in the field of clinical health psychology. The present study examines whether emotional distress and sexual satisfaction mediate the relationship between erectile dysfunction and quality of life and if erectile dysfunction is related to the treatment. For this, a quantitative exploratory design was used. The sample was 44 patients between the ages of 50 to 86 years. It was compiled by availability in different urologists' offices. For its collection, several meetings were held with specialists, a data collection logistics was established, in which the administrative staff identified the participants through their ICD 10 diagnoses. The participants completed four self-administered questionnaires, which most of them showed an appropriate Cronbach's Alpha. The results indicated that exist a low moderately significant relationship between treatment and erectile dysfunction (b = −0.35, 95% BcCI [−0.82, −0.03]) and that there is an indirect effect of psychological distress (b = −0.35, 95 % BcCI [−0.82, −0.03]) and sexual satisfaction (b = −.87, 95% BcCI [−2.18, −0.24] in the relationship between erectile dysfunction and the patient's quality of life. The preliminary data from the present study allow the offering of psychotherapeutic treatments focused on this population.

## RESUMEN

El cáncer de próstata es el más diagnosticado en la población de hombres en Puerto Rico. Sin embargo, es poco estudiado en el campo de la salud, específicamente en el campo de la psicología clínica de la salud. El presente estudio pretende examinar: si la angustia psicológica y la satisfacción sexual median la relación entre disfunción eréctil y calidad de vida, y si la disfunción eréctil se relaciona con el tratamiento que esté recibiendo la persona para tratar su condición. Para esto, se realizó un diseño exploratorio de tipo cuantitativo, con una muestra compuesta por 44 pacientes, entre las edades de 50 a 86 años. La misma fue recopilada por disponibilidad en diferentes oficinas de urólogos. Para su recolección, se realizaron varias reuniones con los especialistas donde se estableció la logística de recogido de datos, en la cual el personal administrativo identificó a los participantes a través de sus diagnósticos según el ICD 10. Los participantes completaron cuatro cuestionarios autoadministrables, los cuales, en su mayoría, mostraron un Alfa de Cronbach apropiado. Los resultados del estudio indican que existe una relación significativa moderada baja entre el tratamiento y la disfunción eréctil (*b* = −0.35, 95%BcCI [−0.82, −0.03]) y que existe un efecto indirecto de la angustia psicológica (*b* = −0.35, 95%BcCI [−0.82, −0.03]) y la satisfacción sexual (*b* = −.87, 95% BcCI [−2.18, −0.24] en la relación entre la disfunción eréctil y la calidad de vida del paciente. Los datos preliminares del presente estudio permiten el ofrecimiento de tratamientos psicoterapéuticos centrados en esta población.

## INTRODUCCIÓN

El cáncer es la enfermedad que causa el mayor número de muertes prematuras en Puerto Rico. En el caso de los hombres, el cáncer de próstata es la primera causa de muerte donde se ha establecido que uno de cada siete va a tener dicha condición en Puerto Rico (Román, 2020). La prevalencia reciente apunta a que el 39.1% de los hombres son diagnosticados anualmente, de los cuales 515 mueren a causa de esta condición (Zavala et al., 2015; Ibarra, 2017). Afortuna-damente existen diversidad de tratamientos para tratar esta condición. El Boletín del Registro de Cáncer del Departamento de Salud de Puerto Rico (2008) y la Sociedad Americana del Cáncer (2015), identifican la prostatectomía radical, la radioterapia y la vigilancia activa, como los tratamientos recomendados por pacientes con cáncer de próstata en estadio inicial. Según la Sociedad Americana del Cáncer (2016), existen otros tipos de tratamientos para estadios más avanzados tales como: la terapia hormonal, la quimioterapia y otros tratamientos inyectables como la vacuna Sipuleucel-T (Provenge) y Eligard (Instituto Nacional del Cáncer, 2020). Si bien las opciones de tratamiento actuales para el cáncer de próstata han resultado ser efectivas en controlar el avance de la condición, y en algunos casos lograr la remisión total, la realidad es que en Puerto Rico la construcción de la masculinidad impacta no tan solo en la detección temprana del diagnóstico, sino también en su tratamiento (temor a los efectos secundarios, entre ellos disfunción eréctil). Por años se ha observado poca apertura a la realización de exámenes de rutina dirigidos a la detección de cáncer de próstata. Esto debido, principalmente, a la construcción de la masculinidad y a la falta de información acerca de los beneficios de la detección temprana, no tan solo vinculada a maximizar años de vida sino también a su sexualidad y a la calidad de vida en general (Selli et al., 2014; Telöken, 2001).

Según la Organización Mundial de la Salud (OMS), el concepto de calidad de vida abarca los siguientes indicadores: a) estatus funcional, el cual mide la presencia y el grado de interferencia de daños físicos en la realización de actividades diarias, de autocuidado, movilidad, actividades físicas propias y cotidianas; b) estatus psicológico, definido por los dominios actuales de regulación emocional, solución de problemas y toma de decisiones; y c) funcionalidad social, referida a redes de apoyo formales. Además, hace referencia a las creencias religiosas de las personas y al funcionamiento global, en el cual se resumen las evaluaciones realizadas por la persona acerca de su bienestar y estado de satisfacción general (Oblitas, 2010).

El cáncer de próstata y su tratamiento afecta de forma general la calidad de vida relacionada con la salud (salud urinaria, sexual y digestiva) de los pacientes, quienes además se enfrentarán a cambios de perspectiva relacionados con su esperanza de vida, integridad corporal y relaciones personales (Sierra et al., 2015). Por ejemplo, a nivel físico el paciente pudiera presentar diversos síntomas tales como: incontinencia urinaria, disfunción intestinal (Sierra et al., 2014) y disfunción eréctil. Esta área en particular definida como la incapacidad de lograr y mantener una erección suficiente para permitir una relación sexual satisfactoria (NHI Consensus Conference, 1993), pudiera provocar sentimientos de vergüenza, incomodidad y la evitación de ciertas conductas conducentes a la intimidad (Sierra et al., 2014). A su vez, el propio diagnóstico pudiera impactar adversamente la salud emocional del paciente si éste no consigue elaborar una respuesta de afrontamiento adecuada a la situación que le permita continuar con el proceso y curso de la enfermedad (Medina & Alvarado, 2011). Esta respuesta puede dar paso a la aparición de la angustia emocional definida como un estado marcado por sentimientos subjetivos, que varían en intensidad desde tristeza, inseguridad, confusión y preocupación, hasta la experiencia de síntomas mucho más severos, por ejemplo, ansiedad, depresión, expresión de la ira, aislamiento social y pérdida de esperanza (Moscoso, 2011). La angustia emocional a su vez puede verse reflejada en estos casos ante la sospecha del diagnóstico de cáncer, el duelo por la pérdida de la salud actual, miedo a la muerte, preocupación por los seres queridos, efectos de los tratamientos y la sobrevivencia o el temor a la recurrencia propia de la condición (Medina & Alvarado, 2011).

Desde la mirada de psicología de la salud resalta la relevancia de evaluar clínicamente la angustia emocional debido a que nos ofrece la oportunidad de identificar pacientes con un elevado riesgo de experimentar alteraciones emocionales antes de ser informados de su diagnóstico de cáncer (Rodgers et al., 2005). Asimismo, evaluaciones de “distrés” emocional posteriores al diagnóstico de cáncer, cirugía o inicio de tratamientos, podrían también ofrecer evidencia clínica valiosa acerca de la relevancia de ofrecer intervenciones de corte psicosocial en diversos escenarios de salud. De esta forma estaríamos abordando la angustia emocional desde un enfoque de intervención temprana y centrado en el paciente con miras a reducir la probabilidad del desarrollo de patologías severas (Andersen et al., 2007).

En el caso de la población con disfunción eréctil producto de los tratamientos recibidos para tratar la condición de cáncer de próstata, la literatura identificada indica, que cuando un hombre experimenta disfunción eréctil suele evitar el contacto y la intimidad sexual (Sousa et al., 2012). De acuerdo a lo reportado, para este no tiene sentido comenzar el contacto sexual pues no podrá completar el acto. Además, estos pacientes afirman que participar en una experiencia sexual pudiera resultar doloroso pues les recuerda su falta de masculinidad, aumentando a su vez sentimientos de angustia psicológica y generando conflictos y frustración con la pareja (Sousa et al., 2012). Para González (2003), la construcción de la masculinidad tradicionalmente se ha basado en el autocontrol de las emociones y los sentimientos con excepción del coraje, y en una concepción estereotipada del hombre. Según Figueroa y Franzoni (2013), la fuerza y poder que se les atribuye a los varones, es la causa de su vulnerabilidad en términos de las prácticas de riesgo para su salud y los mandatos sociales que los someten a mayores cargas de trabajo, a la par que les niegan la capacidad de expresar sus emociones, cuando éstas no corresponden a su rol de género.

Por otra parte, a pesar de que las disfunciones sexuales en oncología son un fenómeno frecuente, se han identificado diversidad de retos entre los que se destacan la pérdida de deseo sexual, la disfunción eréctil y la dispareunia. El origen de estas dificultades pudiera ser no tan solo físico sino también psicológico (Pino, 2010). Ciertamente la sexualidad es considerada actualmente como parte importante e integral del bienestar físico y emocional de las personas; un marcador del estado de salud y calidad de vida (González et al., 2012). Por tanto, merece ser examinada en esta población no desde la óptica comúnmente tratada bajo el modelo biomédico el cual solo requiere que se maneje la enfermedad como una entidad independiente del comportamiento social, trayendo consigo una visión reduccionista (Engel, 1977), sino desde una perspectiva biopsicosocial y centrada en el paciente.

El modelo biopsicosocial sostiene que la interacción de los factores biológicos, psicológicos y sociales pueden ser determinantes de la salud y de la enfermedad (León, 2014). Por tanto, Bishop (1994) define este modelo como un acercamiento sistémico a la enfermedad que enfatiza la interdependencia de estos factores, así como la importancia de abordar la enfermedad en todos sus niveles. Por tal motivo esta investigación tiene como objetivo explorar: 1) si la angustia psicológica media la relación entre la disfunción eréctil y la calidad de vida; 2) si la satisfacción sexual media la relación entre la disfunción eréctil y la calidad de vida; y 3) si la disfunción eréctil se relaciona con el tratamiento que esté recibiendo la persona para tratar su condición.

Ante este planteamiento se formulan las siguientes hipótesis:

H_1_: Existe relación entre la disfunción eréctil con calidad de vida en pacientes con cáncer de próstata en Puerto Rico.

H_2_: La angustia psicológica media la relación entre la disfunción eréctil y la calidad de vida del paciente.

H_3_: La satisfacción sexual media la relación entre la disfunción eréctil y la calidad de vida del paciente.

H_4_: Existe relación entre la disfunción eréctil y el tratamiento en pacientes con cáncer de próstata.

## MÉTODO

### Diseño de Investigación

Este estudio es exploratorio de tipo cuantitativo. Los estudios exploratorios se utilizan para examinar un tema o problema de investigación poco estudiado, del cual se tienen muchas dudas o no se ha estudiado antes (Hernández et al., 2006). Se tomó una muestra no probabilística, la cual fue recopilada por disponibilidad en aquellos pacientes diagnosticados con cáncer de próstata que estuvieran recibiendo tratamiento en oficinas de urólogos al momento de participar en el estudio. Además, la recopilación de los datos se llevó a cabo mediante el autoreporte, en donde la persona completaba los instrumentos de investigación una vez consentía su participación de la investigación.

### Participantes

La muestra del estudio estuvo compuesta por 44 pacientes hombres con cáncer de próstata residentes de Puerto Rico entre las edades de 50 a 86 años (M = 69.53, DE = 7.58). Estos fueron reclutados por disponibilidad en dos oficinas médicas donde se practicaba la urología; una localizada en el área norte y la otra en el área sur de Puerto Rico. El tiempo del diagnóstico de cáncer reportado por los participantes fue desde menos de un año hasta hace 18 años (M = 6.04, DE = 5.82). Poco más de la mitad de los participantes (56.8%, n = 25) indicaron que recibían tratamiento y un 50% (n = 22) indicó que se encontraban en remisión. En cuanto a cuán informados estaban o no del estadio de cáncer, poco más de la mitad (56.8%, n = 25) informó desconocer el estadio en que se encontraba su condición. En cuanto al estado civil, un 61.4%, n = 27) indicó que estaban casados. Mientras que la misma cantidad (61.4%, n = 27) reportó que no trabajaban actualmente. En la Tabla 1, se presentan los datos sociodemográficos reportados por los participantes en su totalidad.

### Instrumentos

#### Cuestionario Sociodemográfico.

El equipo de investigación creó un cuestionario completo con el propósito de obtener información sociodemográfica como edad, estado civil, historial laboral, historial de diagnóstico de cáncer de próstata y tratamientos recibidos.

#### Disfunción Eréctil.

Se utilizó el Cuestionario de Disfunción Eréctil (IIEF 5, International Index Erectile Function) el cual consta de cinco preguntas con cinco opciones de respuesta cada una. El rango de puntuación se encuentra entre 5 y 25 puntos, considerando el punto de corte óptimo en 21. Esta escala posee un alto grado de consistencia interna de .91 (Rosen et al., 1997). Esta escala también ha sido validada en castellano mostrando también adecuadas propiedades psicométricas (Pavía et al., 2012).

#### Calidad de vida.

Con el propósito de medir calidad de vida (CV), se utilizó el cuestionario de la “European Organization for Research and Treatment of Cancer Quality Life” (EORTC -QLQ-C30), el cual es un instrumento específico para examinar el constructo de calidad de vida en poblaciones con cáncer. Está validado para ser implementado en más de 80 idiomas y está compuesto por 30 ítems que valoran la calidad de vida en relación a: aspectos físicos, emocionales y sociales, así como el nivel de funcionalidad de los pacientes diagnosticados con cáncer (Bjordal et al., 2000). El cuestionario se compone a su vez de cinco subescalas. La primera mide funcionalidad a nivel físico, emocional, cognitivo, social y actividades cotidianas. Las próximas tres subescalas examinan síntomas típicos relacionados a los efectos secundarios de los tratamientos (ej., fatiga, dolor y náuseas, vómito) y, la quinta subescala mide el estado global de salud. Este cuestionario tiene a su vez seis ítems independientes (disnea, insomnio, anorexia, estreñimiento, diarrea e impacto económico), que también pueden ser explorados e informan la calidad de vida (Bjordal et al., 2000). En cuanto a las propiedades psicométricas, Arraras et al. (2008) en su estudio, indican que las estimaciones de confiabilidad de consistencia interna estaban por encima de los .70. Además, ha sido traducido y validado al castellano en países latinoamericanos como Colombia, Venezuela, Perú, Chile, Argentina, Costa Rica, Guatemala y Puerto Rico (Soria, et al., 2015).

#### Angustia Psicológica.

Se utilizó la Escala de Malestar Psicológico (K10) de Kessler; escala ampliamente utilizada y traducida en diversos idiomas (árabe, mandarín, alemán, holandés, hebreo, italiano, japonés, portugués y castellano) siendo la traducción al castellano la utilizada en el presente estudio (Brenlla & Aranguren, 2010). Este instrumento mide el riesgo de padecer malestar psicológico inespecífico y consta de 10 reactivos. En cuanto a la consistencia interna de la escala, los valores alfa de Cronbach reportados (.88) son consistentes a los informados por otras investigaciones en diferentes países, donde los coeficientes oscilan entre .84 a .93 (Baggaley et al., 2007; Fassaert et al., 2009; Furakawa et al., 2003; Hides et al., 2007; Kessler et al., 2003). En cuanto a la validez de constructo de la K10, se han observado en estudio previos altas correlaciones con las medidas de depresión y ansiedad (Baggaley et al., 2007; Cairney et al., 2007; Fassaert et al., 2009; Kessler et al., 1993; Schmitz, Lesage & Wang, 2009).

#### Satisfacción Sexual.

Por último, el cuarto instrumento utilizado fue la Nueva Escala de Satisfacción Sexual (NSSS). Esta escala fue traducida al castellano (Pérez, 2013) y diseñada para medir la satisfacción sexual independiente del género, orientación sexual o estatus de relación de pareja (Ahumada et al., 2014). La NSSS examina la satisfacción sexual a través de una escala tipo Likert donde cada pregunta es valorada en el sentido de menor a mayor grado de satisfacción. Pérez (2013), examinó la consistencia interna de la escala traducida al castellano y arrojó un alfa de Cronbach de .93.

Como parte de esta investigación se presentan los resultados de los análisis de consistencia interna de todas las escalas previamente descritas mediante el coeficiente alfa de Cronbach. Si bien los instrumentos contaban con propiedades psicométricas adecuadas para la población hispanohablante, éstos no contaban con un índice psicométrico para la población residente en Puerto Rico. Como parte del presente estudio, los investigadores se dieron a la tarea de también examinar las propiedades psicométricas de estos instrumentos con miras a que pudiesen ser considerados en otras investigaciones y como parte de la práctica clínica.

### Procedimientos Generales

El presente estudio fue aprobado por el Comité de Ética Institucional para la Investigación adscrito a la Universidad donde los autores tienen afiliación. Además, varias reuniones se llevaron a cabo para establecer la logística de recogido de datos en las oficinas de médicos con especialidad en urología con el propósito de salvaguardar la confidencialidad de los participantes y no alterar las operaciones diarias de la práctica. Además, reconociendo los retos que podría traer consigo el reclutamiento de la muestra bajo estudio por la temática a explorar (disfunción eréctil y sexualidad), los médicos recomendaron que la entrega del opúsculo fuera facilitada por el personal administrativo de la clínica como parte de los documentos informativos que constantemente se entregan en una oficina privada y así la relación médico-paciente no se viera afectada de ningún modo. Aquellos potenciales participantes que mostraron interés en participar del estudio tuvieron acceso a la hoja de consentimiento la cual ofreció una descripción detallada acerca del propósito de la investigación, así como el rol voluntario al momento de participar. Además, ofrecía información sobre aspectos de confidencialidad, riesgos asociados a la participación, manejo de eventos en caso de suscitarse algún riesgo y el manejo de los datos recopilados (archivo y destrucción de los materiales confidenciales una vez transcurrido el tiempo requerido por el Comité de Ética). Con el propósito de mantener la confidencialidad del paciente este completó los documentos en una oficina disponible y asignada por el doctor o el personal administrativo correspondiente. La investigadora tomó la certificación de Ley HIPAA y estuvo presente, en un espacio donde se salvaguardó la confidencialidad, los días de recolección de muestra para aclarar cualquier inquietud que se le presentase al potencial participante. El tiempo que tomó el recogido de datos fue de 6 meses. La autora principal fue la encargada principal del recogido de datos.

### Análisis de Datos

Se examinó la confiabilidad para todas las medidas utilizadas en la presente investigación. El criterio para examinar si una prueba o escala posee un nivel de confiabilidad adecuado es poseer un valor alfa de Cronbach de .70 o superior (Field, 2018). Los resultados obtenidos de los instrumentos utilizados fueron codificados y analizados con el programa IBM SPSS versión 25. El criterio para examinar si una prueba o escala posee un nivel de confiabilidad adecuado es poseer un valor alfa de Cronbach de .70 o superior (Field, 2018).

Para conocer si la angustia psicológica y la satisfacción sexual mediaban la relación entre la disfunción eréctil y la calidad de vida, se realizó un análisis de mediación bajo el macro PROCESS para SPSS. Hayes (2018) indica que la mediación es un método estadístico para contestar la pregunta de cómo un agente causal X transmite un efecto en Y. Según el modelo, ocurrirá mediación si la fuerza de la relación entre el predictor (disfunción eréctil) y la variable criterio (calidad de vida) se reduce al incluir al mediador (satisfacción sexual o angustia psicológica). El efecto de este estudio fue evaluado a través del modelo de mediación. Según el modelo, existe un efecto directo si la relación entre la variable predictora y la variable criterio está controlada por la variable mediadora. El efecto es indirecto si la relación entre la variable predictora y la variable criterio es a través de la variable mediadora. El grado de significancia utilizado para esta investigación fue de .05. Para determinar si el efecto indirecto era significativo se utilizó el Intervalo de Confianza con corrección del sesgo a un 95% de probabilidad. Si el Intervalo de Confianza no incluye el cero es un indicativo de que el efecto indirecto no es igual a cero por lo tanto se puede inferir de que estamos frente a una mediación (Hayes, 2018; Kenny, 2018).

## RESULTADOS

### Tratamientos y Efectos Secundarios

A los participantes se les solicitó que indicaran los tratamientos que han recibido para su condición de cáncer de próstata. En esta pregunta los participantes podían seleccionar más de una respuesta por lo que se calculó la frecuencia de respuestas por cada tipo de tratamiento. Casi un 40% de las respuestas fueron dirigidas al tratamiento con radioterapia (38.9%, *n* = 14). Los participantes podían indicar una respuesta diferente a las opciones establecidas por lo que se encontraron las siguientes dos respuestas: Eligard (2.3%, *n* = 1), la cual se utiliza para tratar el cáncer de próstata en estadio avanzado y una inyección sin especificar (2.3%, n = 1). Respecto a los efectos secundarios reportados, más de la mitad de los participantes señalaron presentar disfunción eréctil (53.7%, *n* = 22), seguido por incontinencia urinaria (31.7.3%, *n* = 13) y sensación de cansancio (12.2%, *n* = 5).

### Diagnósticos de Salud Mental y Física

Se les solicitó a los participantes que indicaran si habían sido diagnosticados con algún trastorno de salud mental. Según los resultados obtenidos, casi el 50% de los participantes entrevistados reportaron los diagnósticos de Depresión Mayor (42.9%, *n* = 6) y Ansiedad Generalizada (42.9%, *n* = 6). En cuanto a la salud física, el diagnóstico más reportado fue la hipertensión (71.0%, *n* = 22), seguido de diabetes (16.1%, *n* = 5) y CAD enfermedad coronaria (4.6%, *n* = 2).

### Confiabilidad de las Medidas

Todas las pruebas utilizadas en esta investigación demostraron poseer valores alfa de Cronbach adecuados con excepción de la subescala de fatiga de la escala de Calidad de vida. En la Tabla 6, se presentan todos los valores de confiabilidad alfa de Cronbach para todas las pruebas.

### Análisis de Correlación

Para examinar la relación entre la disfunción eréctil y si la persona recibía o no tratamiento se utilizó una prueba de punto biserial. El resultado obtenido indica que existe una relación significativa moderada baja (Champion, 1981) entre las variables. Cabe mencionar que al ser un análisis de punto biserial y las respuestas de sí y no, estaban codificadas como 1 para sí y 0 para no, por lo que el resultado obtenido (*rbp* = −.34, *p* < .05) indica que las puntuaciones altas en disfunción eréctil se asocian con las personas que no recibieron tratamiento. De los que no recibieron tratamiento para el cáncer, 33.3% (*n* = 7) padecen de alguna condición crónica. La hipertensión fue la condición de salud más reportada. El resultado obtenido para la correlación Spearman rho para la calidad de vida y la disfunción eréctil fue de *rho* = −.12, *p* = .46, mientras el resultado de la correlación de punto biserial para la disfunción eréctil y si la persona recibe o no tratamiento es de *I* = −.34, *p* < .05.

### Análisis de Mediación

**Figura 1.**
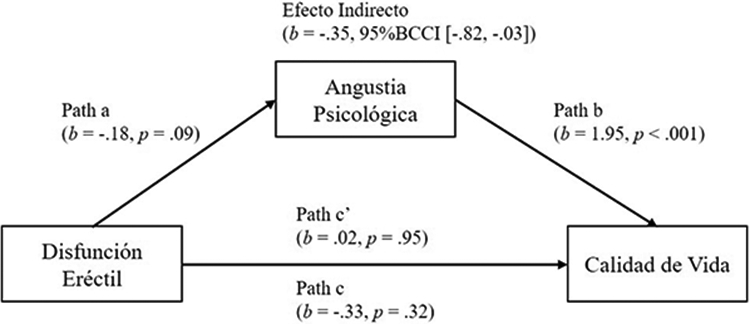
Rol Mediador de la Angustia Psicológica en la Relación Entre la Disfunción Eréctil y la Calidad de Vida.

Se realizó un análisis de mediación para examinar el rol mediador de la angustia psicológica en la relación entre la disfunción eréctil y la calidad de vida del paciente. Los resultados obtenidos indican que el modelo entre la disfunción eréctil y la angustia psicológica (path a) no tiene un buen ajuste (*F*(1, 36) = 3.07, *p* = .09), explicando un 8% de la varianza, y en donde la disfunción eréctil no predice la angustia psicológica significativamente (*b* = −.18, *p* = 0.09). En cuanto al modelo del path b y c’ se encontró que tiene un buen ajuste (*F*(2, 35) = 11.36, *p* < .001), explicando un 39% de la varianza, y en donde la angustia psicológica (path b) predice la calidad de vida significativamente (*b* = 1.95, p < .001), y la disfunción eréctil (path c’) no predice significativamente la calidad de vida (b = .02, p = .95) cuando la angustia psicológica está en el modelo. Al examinar el efecto total (path c) se pudo observar que el modelo no logra un buen ajuste (*F*(1, 36) = 1.03, *p* = .32), explicando un 3% de la varianza, en donde la disfunción eréctil no logra predecir a la calidad de vida (*b* = −.33, *p* = .32) cuando la angustia psicológica no está en el modelo. Finalmente, se pudo observar un efecto indirecto de la angustia psicológica en la relación entre la disfunción eréctil y la calidad de vida del paciente (*b* = −0.35, 95%BcCI [−0.82, −0.03]). Este resultado indica que la angustia psicológica media completamente la relación entre la disfunción eréctil y la calidad de vida. La Figura 1 muestra el modelo de mediación puesto a prueba con sus respectivos valores.

Además, se realizó un análisis de mediación para examinar el rol mediador de la satisfacción sexual en la relación entre la disfunción eréctil y la calidad de vida del paciente. Los resultados obtenidos indican que el modelo entre la disfunción eréctil y la satisfacción sexual (path a) tiene un buen ajuste (*F*(1, 33) = 33.70, *p* < .001), explicando un 51% de la varianza, y en donde la disfunción eréctil predice la satisfacción sexual significativamente (*b* = 2.12, *p* < .001). En cuanto al modelo del path b y c’, se encontró que tiene un buen ajuste (*F*(2, 32) = 4.08, *p* < .05), explicando un 20% de la varianza, y en donde la satisfacción sexual (path b) predice la calidad de vida significativamente (b = −.41, *p* < .05), y la disfunción eréctil (path c’) no predice significativamente la calidad de vida (*b* = .56, *p* = .23) cuando la satisfacción sexual está en el modelo. Al examinar el efecto total (path c) se pudo observar que el modelo no logra un buen ajuste (*F*(1, 33) = 0.80, *p* = .38), explicando un 2% de la varianza, en donde la disfunción eréctil no logra predecir a la calidad de vida (*b* = −.31, *p* = .38) cuando la satisfacción sexual no está en el modelo. Finalmente, se pudo observar un efecto indirecto de la satisfacción sexual en la relación entre la disfunción eréctil y la calidad de vida del paciente (*b* = −.87, 95% BcCI [−2.18, −0.24]. Este resultado indica que la satisfacción sexual media completamente la relación entre la disfunción eréctil y la calidad de vida

## DISCUSIÓN

El cáncer de próstata es una de las principales causas de muerte en varones residentes en Puerto Rico (Zavala et al., 2015) y una condición que impacta la calidad de vida del paciente (Sierra et al., 2014). Las opciones para el tratamiento dependen de varios factores entre ellos: individuales (la salud física y emocional en general), contextuales y sociales, así como aquellos relacionados al estadio de la condición. También se evalúan los beneficios y costos asociados a recibir un tratamiento por otro (Mayo Clinic, 2018). Entre los efectos secundarios más notables, está la disfunción eréctil. Muchos hombres pueden tener dificultad para obtener o mantener erecciones después de varias formas de tratamiento contra el cáncer (Metz, 2018). Los estudios sobre la calidad de vida de hombres con cáncer de próstata generalmente se han enfocado primordialmente en los efectos secundarios y en los síntomas físicos asociados a su tratamiento (Berrios & Rivero, 2015). Sin embargo, otros aspectos psicosociales de la enfermedad no han recibido la misma atención (Song, 2009) trayendo consigo que el campo de psicología clínica de la salud cobre aún mayor pertinencia. El presente estudio está sustentado bajo el modelo biopsicosocial, que sostiene la relevancia de estudiar la condición y prestarle servicios desde la interacción de factores biológicos, psicológicos y sociales desde una perspectiva centrada en el paciente. Por tanto, esta investigación tuvo como propósito investigar: 1) si la disfunción eréctil en pacientes con cáncer de próstata se relaciona con la calidad de vida; 2) si la angustia psicológica media la relación entre disfunción eréctil y calidad de vida; 3) si la satisfacción sexual media la relación entre la disfunción eréctil y calidad de vida; 4) averiguar si la disfunción eréctil se relaciona con el tratamiento.

Cuando se examinó el efecto mediador de la angustia psicológica, los resultados obtenidos sugieren que media completamente la relación entre la disfunción eréctil y la calidad de vida del paciente. Esto significa que la disfunción eréctil afecta la salud emocional produciendo angustia psicológica o distress en el paciente con cáncer de próstata, lo que a su vez afecta su calidad de vida. Si bien se ha documento en la literatura cómo la disfunción eréctil es causada por una amplia gama de factores orgánicos, psicológicos, psiquiátricos, interpersonales y farmacológicos (González et al., 2012), este estudio añade una variable a este complejo escenario, la angustia emocional. Resulta necesario no tan solo reconocer el efecto que tiene la disfunción sexual en la calidad de vida de esta población, sino cómo la evaluación de la angustia emocional nos puede ofrecer la oportunidad de dirigir intervenciones psicológicas que puedan atender los síntomas asociados y así evitar que estos síntomas afecten el bienestar de la persona con el diagnóstico (Andersen et al., 2007; Oraa et al., 2013).

Según se mencionó previamente, uno de los objetivos de la presente investigación era examinar si la satisfacción sexual mediaba la relación entre la disfunción eréctil y calidad de vida. Según los hallazgos, la satisfacción sexual media completamente la relación entre estas variables lo cual sugiere que la disfunción eréctil afecta la satisfacción sexual del paciente con cáncer de próstata, lo que su vez afecta su calidad de vida. Dado que la disminución sexual, la pérdida de placer y la disminución de la capacidad para actividad sexual son consecuencias asociadas al tratamiento para el cáncer de próstata, y que luego del tratamiento muchos hombres y su parejas presentan disminución sexual en respuesta a la disfunción eréctil (Wittmann et al., 2009), dicha información ofrece la oportunidad tanto a psicólogos de la salud como a personal médico de abordar este tema con los pacientes antes, durante y una vez finalizado el tratamiento para el manejo de la condición (Twitchell et al., 2019). También resulta relevante que como parte de nuestro abordaje clínico podamos realizar intervenciones psicológicas dirigidas a una redefinición de la sexualidad (Martínez et al., 2020) que faciliten mayor bienestar sexual tanto del paciente como el de su pareja, con el propósito de promover una vida sexual saludable que mejore tanto su calidad de vida como su bienestar en general (NIH, 2013).

Por último, esta investigación deseaba comprobar si existía o no relación entre la disfunción eréctil y el tratamiento en pacientes con cáncer de próstata. Los resultados obtenidos sugieren que existe una asociación moderada baja entre la disfunción eréctil y el tratamiento. No obstante, los resultados obtenidos en el análisis biserial indican que las puntuaciones altas en disfunción eréctil se asocian con las personas que no recibieron tratamiento. Si bien la literatura señala que el tratamiento para el cáncer de próstata pudiera estar asociado con disfunción eréctil, sabemos que éste pudiera deberse a multiplicidad de factores que merecen se les preste mayor estudio. Por ejemplo, la mayoría de los participantes de este estudio reportaron padecer de hipertensión y diabetes. En el caso de personas con hipertensión, se ha encontrado que algunos medicamentos antihipertensivos, especialmente los diuréticos y beta bloqueadores (Mas, 2005) impactan dicha área. En el caso de los hombres con diabetes, se ha encontrado que lesiones en los nervios y en los vasos sanguíneos causadas por un control deficiente de los niveles de azúcar en sangre a largo plazo (Mayo Clinic, 2018) también incide en la disfunción eréctil. Esto quiere decir, que los resultados elevados en la escala de disfunción eréctil pudieran estar influenciados por otras condiciones de salud adicionales a su diagnóstico de cáncer de próstata o con algún otro tratamiento que esté recibiendo la persona para tratar otras afecciones de salud.

### Limitaciones

La investigación presenta limitaciones en términos del muestreo utilizado, el cual fue por disponibilidad. Esto limita la capacidad de generalización de los datos obtenidos. Es importante mencionar que, a pesar del tamaño de la muestra, fue posible probar los objetivos del estudio y realizar los análisis establecidos originalmente, por lo cual, esta investigación ofrece información preliminar y relevante acerca de la mediación de la angustia psicológica y la satisfacción sexual del hombre que padece de disfunción eréctil con el propósito de examinar estas áreas en esta población y poder ofrecer tratamientos que sean responsivos y centrados a sus necesidades. Esto con el fin ulterior de mejorar su calidad de vida. Si bien no se observó un patrón definido de datos sin contestar (*missing values*), ciertamente la recopilación de datos mediante autoreporte y en papel trae consigo dicho reto pues no se pudieron identificar en el momento las preguntas sin contestar. Otra limitación identificada en el presente estudio fueron los terremotos ocurridos en Puerto Rico entre diciembre 2019 y enero 2020. Dichos fenómenos naturales pudieron haber alterado las puntuaciones de la escala de distress o angustia emocional ya que parte de la muestra fue obtenida en uno de los pueblos más afectados.

Para futuras investigaciones se exhorta, considerar realizar comparaciones entre el efecto mediador de la angustia psicológica y la disfunción eréctil por estadios del cáncer o agruparlos entre estadio I y II y estadio III y IV con el propósito de examinar si varían por grupos y así realizar intervenciones psicoterapéuticas más individualizadas según la etapa en la que se encuentre la persona. Para ello, será necesario también evaluar la comunicación entre médicos y pacientes la cual la literatura de por sí ha reportado que suele ser un vínculo complejo y que informa no tan solo el quehacer médico, sino que es la base de una adecuada práctica de la disciplina médica (Meza et al., 2013). En este estudio pudimos observar cómo más de la mitad de los participantes reportaron desconocer el estadio de su condición. Otras variables a considerarse en futuros estudios pudieran ser, por ejemplo, el tiempo que lleva el paciente con el diagnóstico y el efecto que podrían tener las variables mediadoras a través del tiempo. Esto con el interés de identificar el impacto de la enfermedad tanto a nivel sexual como emocional y poder realizar intervenciones a nivel preventivo.

## CONCLUSIÓN

Los resultados obtenidos en esta investigación sugieren que tanto la angustia psicológica como la satisfacción sexual median completamente la relación entre la disfunción eréctil y la calidad de vida en paciente adultos diagnosticados con cáncer de próstata residentes en Puerto Rico. Este conocimiento nos permite describir cómo pudiera afectar a los hombres la disfunción eréctil, también identificar sus necesidades y estrategias de afrontamiento para realizar un plan de cuidado compuesto por un equipo interdisciplinario, proporcionando atención al paciente tanto a nivel ambulatorio como hospitalario (Franquet et al., 2011). Parte de los esfuerzos del campo de psicología de salud es poder promover no tan solo colaboración de las diversas disciplinas en el campo de la salud sino también facilitar su integración en las áreas de promoción, prevención y, como en este caso, de tratamiento y manejo de la condición.

Los datos de esta investigación a su vez contribuyen al desarrollo de nuevas investigaciones sobre el impacto que tiene la disfunción eréctil en la salud mental y sexual de los pacientes que padecen de cáncer de próstata en Puerto Rico. Diversos autores destacan la importancia de realizar investigaciones sobre los aspectos psicosociales para la recuperación sexual después del cáncer de próstata (Song, 2009). Además, puede contribuir a la creación de programas de psicología de la salud para tratar a los pacientes antes y durante su tratamiento incluyendo a sus parejas. El uso de escalas, como las que se utilizaron en este estudio, puede ser de gran utilidad a los profesionales de la salud que atienden a pacientes con cáncer de próstata y padecen de disfunción eréctil. Los hallazgos obtenidos muestran que dichos instrumentos cuentan con propiedades psicométricas óptimas los cuales podrán ser útiles para identificar la salud emocional, sexual y calidad de vida del paciente y de esa manera poder ofrecer intervenciones adecuadas y responsivas al paciente.

Por último, es importante resaltar que este estudio es pionero en Puerto Rico ya que se examina el tema de la sexualidad y la identificación de aspectos emocionales en una población casi invisibilizada. Si bien se conoce que esta condición es una de las principales causas de muerte entre hombres, poco se habla de ella y las campañas de prevención son casi inexistentes contrario a lo que se observa con el cáncer de mama. El objetivo de este estudio, además de contar con datos preliminares acerca de la relación entre la sexualidad y variables de salud emocional y cómo éstas incidían en la calidad de vida, promueve que se pueda contar con un punto de partida al momento de ofrecer tratamientos psicoterapéuticos a esta población. Se requiere entonces que esta condición crónica pueda ser abordada y tratada desde un enfoque biopsicosocial donde se atienda su vez la masculinidad, la sexualidad y la salud emocional de esta población. Confiamos que el presente estudio pueda ser el primero de muchos dedicados a mejorar la calidad de vida de los pacientes con cáncer de próstata en Puerto Rico.

## Figures and Tables

**Tabla 1:** Datos Sociodemográficos (n = 44)

	*f*	%
*Recibe Tratamiento*		
Sí	25	56.8
No	13	29.5
Missing	6	13.6
*Remisión*		
Sí	22	50.0
No	11	25.0
Missing	11	25.0
*Estado Civil*		
Soltero	5	11.4
Casado	27	61.4
Divorciado	3	6..8
Convive	2	4.5
Viudo	3	6.8
Missing	4	9.1
*Trabajo*		
Sí	14	31.8
No	27	61.4
Missing	3	6.8
*Estadio de Cáncer*		
I	4	9.1
II	0	0.0
III	3	6.8
IV	2	4.5
Desconoce	25	56.8
Missing	10	22.7

*Nota. f* = frecuencia; % = porciento.

**Tabla 2:** Alfa de Cronbach por Escalas

	*α*
**Calidad de Vida (Escala Total)**	.85
Estatus de Salud Global	.96
Función Física	.84
Función de Rol	.87
Función Emocional	.89
Función Cognitiva	.72
Función Social	.79
Fatiga	.63
Náusea / Vómitos	1.0
Dolor	.80
Kessler	.87
Disfunción Eréctil (*Salud Sexual*)	.96
Nueva Escala de Satisfacción Sexual	.99

*Nota. α* = Coeficiente alfa de Cronbach.

## References

[R1] Abdulkhaleq A, Ghalib A, Naqeeb A, & Aziz A (2017). Evaluation of prostate cancer health- related quality of life (specific scale) among a sample in Baghdad City. World Journal of Pharmaceutical Research 6(8), 61–75. https://1library.net/document/yr34mnpy-evaluation-prostate-cancer-health-related-quality-specific-baghdad.html

[R2] Ahumada S, Lüttges C, Molina T, & Torres S (2014). Satisfacción sexual: Revisión de los factores individuales y de pareja relacionados. Rev. Hosp Clín Univ Chile, 25, 278–284. https://www.enfermeriaaps.com/portal/wp-content/uploads/2017/04/Satisfacción-sexual.-Revisión-de-los-factores-individuales-y-de-pareja-relacionados.pdf

[R3] Andersen BL, Farrar WB, Golden D, Emery CF, Glaser R, Crespin T (2007). Distress reduction from a psychological intervention contributes to improved health for cancer patients. Brain, Behavior, and Immunity, 21(7), 953–961. 10.1016/j.bbi.2007.03.005 PMC2039896 17467230

[R4] Arraras J, Villafranca E, Arias F, Dominguez M, Lainez N, Manterola A, Martínez E, Romero P, & Martínez M (2008). The EORTC Quality of Life Questionnaire QLQ-C30 (version 3.0). Validation study for Spanish prostate cancer patients. Archivos Españoles de Urología, 6,949–954. 10.4321/s0004-06142008000800017 19040169

[R5] Asociación Española Contra el Cáncer. (junio de 2011). Comunicación y Apoyo. https://www.aecc.es/SobreElCancer/CancerPorLocalizacion/CancerMama/viviendodiaadia/vidaenpareja/Paginas/sexualidad.aspx

[R6] Baggaley RF, Ganaba R, Filippi V, Kere M, Marshall T, Sombié I (2007). Detecting depression after pregnancy: The validity of the K10 and K6 in Burkina Faso. Tropical Medicine & International Health, 12, 1225–1229. 10.1111/j.1365-3156.2007.01906.x 17956505

[R7] Berríos R, & Rivero A (2015). El cáncer prostático en la experiencia de pareja: La mujer como guardiana de la sa-lud. Interamerican Journal of Psychology, 49, 387–398. 10.30849/rip/ijp.v49i3.87

[R8] Bishop GD (1994). Healthy psychology: Integrating mind and body. Allyn and Bacon.

[R9] Bjordal K, de Graeff A, Fayers PM, Hammerlid E, van Pottels-berghe C, Curran D, Ahlner-Elmqvist M, Maher EJ, Meyza JW, Brédart A, Söderholm AL, Arraras JJ, Feine JS, Abend-stein H, Morton RP, Pignon T, Huguenin P, Bottomly A, & Kaasa S (2000). A 12 country field study of the EORTC QLQ-C30 (version 3.0) and the head and neck cancer specific module (EORTC QLQ-H&N35) in head and neck patients. European Journal of Cancer, 36, 1796–1807. 10.1016/s0959-8049(00)00186-6 10974628

[R10] Brenlla ME, & Aranguren M (2010). Adaptación argentina de la escala de malestar psicológico de Kessler (K10). Revista de Psicología, 28, 311–342. http://revistas.pucp.edu.pe/index.php/psicologia/article/view/1464/1411

[R11] Cairney J, Veldhuizen S, Wade T, Kurdyak P, & Streiner D (2007). Evaluation of 2 measures of psychological distress as screeners for depression in the general population. Canadian Journal of Psychiatry, 52, 111–120. 10.1177/070674370705200209 17375867

[R12] Champion DJ (1981). Basic statistics for social research (2^nd^ ed.). McMillan

[R13] Departamento de Salud de Puerto Rico. (2008). Boletín del Registro de Cáncer. Registro Central de Cáncer de Puerto Rico, 1, 1–7.

[R14] Engel GL (1977). The need for a new medical model: A challenge for biomedicine. Science, 196, 129–136. 10.1126/science.847460 847460

[R15] Fassaert T, De Wit M, Tuinebreijer W, Wouters H, Verhoeff A, Beekman A (2009). Psychometric properties of an interviewer-administered version of the Kessler Psychological Distress scale (K10) among Dutch, Moroccan and Turkish respondents. International Journal of Methods in Psychiatric Research, 18, 159–168. 10.1002/mpr.288 19701920 PMC6878421

[R16] Figueroa JG, & Franzoni J (2013). Políticas públicas, varones y equidad de género: El uso de México dentro de una búsqueda multinacional. http://www.lazoblanco.org/wpcontent/uploads/2013/08manual/bibliog/material_masculinidades_0054.pdf

[R17] Figueroa NR, De La Torre T, Torres M, Pérez J, Ortiz KJ, Calo W, Santana M, & Rodríguez S (2008). Cáncer en los hombres. Boletín Registro de Cáncer de Puerto Rico, 1(3), 1–8. https://estadisticas.pr/files/Inventario/publicaciones/DS_BoletinVol1Num3CancerenlosHombres_2008_0.pdf

[R18] Franquet E, García C, Navarro L, Palomino A, Panisello N, & Pardes V (2011). ¿Cómo afrontan los hombres la disfunsión eréctil?. Asociación Española de Enfermería en Urología, 118(abril/mayo/junio), 20–28.

[R19] Furukawa T, Kessler R, Slade T & Andrews G (2003). The performance of the K6 and K10 screening scales for psychological distress in the Australian National Survey of Mental Health and Well-Being. Psychological Medicine, 33, 357–362. 10.1017/s0033291702006700 12622315

[R20] González J (2003). Construcción, cuestionamiento y reconstrucción del concepto masculinidad. Género, sociedad y cultura, 43–63.

[R21] González EG, Lorenzo O, & De La Paz Y (2012). Cáncer de próstata y sexualidad. Acta Médica del Centro, 6, 102–105. http://www.actamedica.sld.cu/r2_12/pdf/prostata.pdf

[R22] Hayes AF (2018). Introduction to mediation, moderation, and conditional process analysis: A regression-based approach. The Guilford Press.

[R23] Hernández R, Fernández C, & Baptista P (2006). Metodología de la Investigación. McGraw-Hill Interamericana.

[R24] Hides L, Lubman DI, Devlin H, Cotton S, Aitken C, Gibbie T (2007). Reliability and validity of the Kessler 10 and Patient Health Questionnaire among injecting drug users. Australian and New Zealand Journal of Psychiatry, 41, 166–168. 10.1080/00048670601109949 17464695

[R25] Ibarra G (2017, febrero). La sombra del cáncer y su impacto en Puerto Rico. CB en Español. https://cb.pr/la-sombra-del-cancer-y-suimpacto-en-puerto-rico/?cn-reloaded=1

[R26] Instituto Nacional de Cáncer. (2020,). Diccionario de cáncer. https://www.cancer.gov/espanol/publicaciones/diccionario/def/eligard

[R27] Kenny DA (2018). Mediation. http://davidakenny.net/cm/mediate.htm

[R28] Kessler R, Barker P, Colpe L, Epstein J, Gfroerer J, Hiripi E (2003). Screening for serious mental illness in the general population. Archives of General Psychiatry, 60(2), 184–189. 10.1001/archpsyc.60.2.184 12578436

[R29] León JM (2014, septiembre). Fundamentos de la psicología de la salud. http://openaccess.uoc.edu/webapps/o2/bitstream/10609/78524/3/Psicolog%C3%ADa%20de%20la%20salud%20y%20calidad%20de%20vida_M%C3%B3dulo%201._Fundamentos%20de%20la%20psicolog%C3%ADa%20de%20la%20salud.pdf

[R30] Martínez A, Fernández C, Puga AP, López OM, Lucas M, Granero J, Fernández IM & Hernández JM (2020). Sexual experiences after non-nerve sparing radical prostatectomy. 10.37689/acta-ape/2020ao02375

[R31] Mas M (2005). Hipertensión arterial, medicación antihipertensiva y disfunción eréctil una perspectiva basada en la evidencia. Revista Internacional de Andrología: Salud sexual y reproductiva, 3, 13–30. 10.1016/S1698-031X(05)74684-7

[R32] Mayo Clinic. (2018, noviemebre). Disfunción eréctil y diabetes: Toma el control hoy. https://www.mayoclinic.org/eses/diseases-conditions/erectile-dysfunction/in-depth/erectiledysfunction/art-20043927

[R33] Medina X, & Alvarado S (2011). Psicooncología: Una respuesta al malestar emocional del paciente oncológico. Healthcare Journal of Medicine, 3(2), 190–196. https://www.uroclinic.com.ec/psicooncologia-una-respuestaal-malestar-emocional-del-paciente-oncologico/

[R34] Metz J (2018). Disfunción erectil después del tratamiento del cáncer. OncoLink. https://es.oncolink.org/apoyar/sexualidad-y-fertilidad/sexualidad/disfuncion-erectil-despues-del-tratamiento-del-cancer

[R35] Meza MP, Sánchez C, & Mancilla J (2013). Relación médico-paciente con cáncer. http://www.scielo.org.mx/scielo.php?script=sci_arttext&pid=S0187-53372014000100007

[R36] Moscoso M (2011). El estrés crónico y la medición psicométrica del distrés emocional en medicina y psicología de la salud. Liberabit, 17, 67–76. http://www.scielo.org.pe/pdf/liber/v17n1/a08v17n1.pdf

[R37] National Institutes of Health. (1993). Impotence [consensus development panel]. Journal of the American Medical Association, 270, 83–90. 10.1001/jama.1993.035100100890368510302

[R38] Oblitas L (2010). El cáncer de próstata localizado, la calidad de vida y el ajuste marital . Cengage Learning. https://books.google.com.pr/books?id=LXR_dkV_XNcC&pg=PA550&lpg=PA550&dq

[R39] Oraa N, Sánchez M, Ossola G, Vélez E, Cevas FJ, & del Pino N. (2013). Eficacia de las intervenciones psicológicas en hombres con cáncer de próstata. Psicooncolgía, 10, 339–351. 10.5209/rev_PSIC.2013.v10.n2-3.43454

[R40] Pavía N, López N, & Vera L (2012). Disfunción eréctil en pacientes con enfermedades crónico degenerativas y metabólicas en una población rural de Yucatán, México. Revista Mexicana de Urología, 72(5), 240–244. https://www.elsevier.es/es-revista-revista-mexicana-urologia-302-pdf-X2007408512679435

[R41] Pérez F (2013). Nueva escala de satisfacción sexual (NSSS) en usuarios de redes sociales [Trabajo fin de Master, Universidad de Almería]. Archivo digital. http://repositorio.ual.es/bitstream/handle/10835/2366/Trabajo.pdf?sequence=1&isAllowed=y

[R42] Pino CE (2010). Disfunción sexual en pacientes con cáncer. Revista de los Estudiantes de Medicina de la Universidad Industrial de Santander, 23, 126–133. https://revistas.uis.edu.co/index.php/revistamedicasuis/article/view/1441/1844

[R43] Registro Central de Cáncer. (2010). Incidencia de cáncer en Puerto Rico. Departamento de Salud Gobierno de Puerto Rico. http://www.salud.gov.pr/Estadisticas-Registros-y-Publicaciones/Pages/Registros/Registro-de-C%C3%A1ncer.aspx

[R44] Rivera & Berrio (2016). El cáncer de próstata y la construcción social de la masculinidad en Puerto Rico. Psicología, Conocimiento y Sociedad 6, 164–190.

[R45] Rodgers J, Martin C, Morse R, Kendell K & Verrill M (2005). An investigation into the psychometric properties of the Hospital Anxiety and Depression Scale in patients with breast cancer. Health and Quality of Life Outcomes, 3, 41–53. 10.1186/1477-7525-3-41 16018801 PMC1184094

[R46] Román W (2020, junio). Uno de cada siete hombres tendrá cáncer de próstata en Puerto Rico. MSP Medicina Salud Pública. https://medicinaysaludpublica.com/uno-de-cada-siete-hombres-tendra-cancer-de-prostata-en-puerto-rico/

[R47] Rosen RC, Riley A, Wagner G, Osterloh IH, Kirkpatrick J, & Mishra A (1997). The international index of erectile function (IIEF): a multidimensional scale for assessment of erectile dysfunction. Urology, 49(6), 822–830. 10.1016/s0090-4295(97)00238-0 9187685

[R48] Schmitz N, Lesage A & Wang JL (2009). Should psychological distress screening in the community account for self-perceived health status? Canadian Journal of Psychiatry 54(8), 526–533. 10.1177/070674370905400805 19726005

[R49] Selli C, Bjartell A, Burgos J, Somerville M, Palacios JM, Benjamín L, Black L, & Castro R (2014). A One-Year, Pan-European Observational Study. Hindawi Publishing Corporation Prostate Cancer. 10.1155/2014/472949 PMC3976870 24757567

[R50] Sierra CR, Sánchez D, & de Pablo A (2015). Calidad de vida de pacientes con cáncer de próstata en tratamiento con bloqueo androgénico continuo vs intermitente: Estudio prospectivo mediante la aplicación del cuestionario CAVIPR. Anales del Sistema Sanitario de Navarra, 38(2), 193–201. http://scielo.isciii.es/pdf/asisna/v38n2/original2.pdf 26486525 10.23938/ASSN.0068

[R51] Sierra KL, Viveros C, Martínez G, Hernández O, & Caballero G (2014). Calidad de vida en pacientes con cáncer de próstata, operados de Prostatectomía radical laparoscópica. Revista Mexicana de Urologia, 74(3), 133–140. 10.1016/S2007-4085(15)30027-6

[R52] Sociedad Americana del Cáncer. (2015). ¿Qué indican las estadísticas clave sobre el cáncer prostático?. http://www.cancer.org/espanol/cancer/cancerdeprostata/guiadetallada/cancer-deprostata-what-is-key-statistics

[R53] Sociedad Americana del Cáncer. (2016, Febrero). Qué es el Cancer de prostata?. Sociedad Americana del Cáncer. https://www.cancer.org/es/cancer/cancer-de-prostata/acerca/que-es-cancer-de-prostata.html#referencias

[R54] Song L (2009). Couples communication and quality of life during prostate Cancer survivorship. [Unpublished Dissertation]. The University of Michigan, Michigan.

[R55] Soria JM, Carrasco JG, Loza C, Ruíz E, & Payet E (2015). Adaptación cultural y validación psicométrica del cuestionario de calidad de vida EORTC QLQ STO-22 para los pacientes con cáncer gástrico en el Perú. Revista Gastroenterología en Perú, 35, 127–136. 26228978

[R56] Sousa AD, Sonavane S, & Metha J (2012). Psychological aspects of prostate cancer: a clinical review. Prostate cancer and Prostatic Disease, 15, 120–127. 10.1038/pcan.2011.66 22212706

[R57] Telöken C (2001). Management of erectile dysfunction secondary to treatment for localized prostate cancer. Cancer Control, 8, 540–545. 10.1177/107327480100800609 11807424

[R58] Twitchell DK, Wittmann DA, Hotaling JM, & Pastuszak AW (2019). Psychological impacts of male sexual dysfunction in pelvic cancer survivorship. Sex Med,7, 614–626. 10.1016/j.sxmr.2019.02.003 PMC6763375 30926459

[R59] Wittmann D, Northouse L, Foley S, Gilbert S, Wood DP, Balon R, & Montie JE (2009). The psychosocial aspects of sexual recovery after prostate cancer treatment. International Journal of Impotence Research, 21, 99–106. 10.1038/ijir.2008.66 19158798

[R60] Zavala D, Tortolero G, Torres CR, Alvarado M, Traverso M, Román Y, & Ortiz KJ (2015). Cáncer en Puerto Rico, 2008-2012. Registro Central de Cáncer de Puerto Rico.

